# Characterizing Aeroallergens by Infrared Spectroscopy of Fungal Spores and Pollen

**DOI:** 10.1371/journal.pone.0124240

**Published:** 2015-04-13

**Authors:** Boris Zimmermann, Zdenko Tkalčec, Armin Mešić, Achim Kohler

**Affiliations:** 1 Department of Mathematical Sciences and Technology, Norwegian University of Life Sciences, Ås, Norway; 2 Division for Marine and Environmental Research, Ruđer Bošković Institute, Zagreb, Croatia; University of Wisconsin—Madison, UNITED STATES

## Abstract

**Background:**

Fungal spores and plant pollen cause respiratory diseases in susceptible individuals, such as asthma, allergic rhinitis and hypersensitivity pneumonitis. Aeroallergen monitoring networks are an important part of treatment strategies, but unfortunately traditional analysis is time consuming and expensive. We have explored the use of infrared spectroscopy of pollen and spores for an inexpensive and rapid characterization of aeroallergens.

**Methodology:**

The study is based on measurement of spore and pollen samples by single reflectance attenuated total reflectance Fourier transform infrared spectroscopy (SR-ATR FTIR). The experimental set includes 71 spore (Basidiomycota) and 121 pollen (Pinales, Fagales and Poales) samples. Along with fresh basidiospores, the study has been conducted on the archived samples collected within the last 50 years.

**Results:**

The spectroscopic-based methodology enables clear spectral differentiation between pollen and spores, as well as the separation of confamiliar and congeneric species. In addition, the analysis of the scattering signals inherent in the infrared spectra indicates that the FTIR methodology offers indirect estimation of morphology of pollen and spores. The analysis of fresh and archived spores shows that chemical composition of spores is well preserved even after decades of storage, including the characteristic taxonomy-related signals. Therefore, biochemical analysis of fungal spores by FTIR could provide economical, reliable and timely methodologies for improving fungal taxonomy, as well as for fungal identification and monitoring. This proof of principle study shows the potential for using FTIR as a rapid tool in aeroallergen studies. In addition, the presented method is ready to be immediately implemented in biological and ecological studies for direct measurement of pollen and spores from flowers and sporocarps.

## Introduction

Fungal spores and plant pollen are ever-present components of the Earth’s atmosphere. They have long been known to cause respiratory diseases in susceptible individuals, such as asthma, allergic rhinitis and hypersensitivity pneumonitis. These diseases affect 10–20% of the world population (they are higher in temperate and lower in tropic zones), and causing multibillion costs of medical treatments and reduced work productivity [1,2]. A number of epidemiological studies show that in the last two decades the prevalence of respiratory and atopic allergies has been increasing [3–5]. Furthermore, climate change is likely to result in substantial weather variations, such as temperature, humidity and precipitation, thus shifting the production and distribution of allergenic pollen and spores. The impacts of allergic diseases on public health, and thus economy, are enormous. It is expected that shifts in aeroallergen human exposures due to global climate change are going to increase the prevalence and severity of allergies [6]. In general, allergies cannot be cured, but symptoms can be reduced by medical treatment before pollen exposure. Therefore, aeroallergen monitoring networks performing qualitative and quantitative analysis of bioparticles are an important part of treatment strategies. Providing information on their occurrence in residential and working areas is important for timely treatment of allergies through symptoms correlation.

In contrast to pollen, the role of fungi as a driver of respiratory allergies has not been sufficiently explored. This is unfortunate given the fact that fungal spores have been causing more severe allergic reactions than pollen, such as severe asthma with fungal sensitization [7,8]. Moreover, the number of fungal spores per cubic metre of air can often exceed pollen concentrations by three orders of magnitude [9]. Understanding the role of fungal exposure has been limited because fungal spores are extremely hard to identify, while their atmospheric distribution and concentration is highly variable. Spore dispersal is not only dependent on the weather variables, such as wind and rainfall, but also on the circadian rhythm, growth and distribution of fungi that produce them [10,11]. A recent study of airborne fungal spores, based on a molecular approach, has found that over 86% of genera detected by DNA sequencing were not routinely identifiable by traditional microscopy analysis [12]. In addition, microscopic analysis of fungal spores is very time consuming, and in most cases cannot ensure identification to the species or genus level, due to the great number of species and genera with morphologically very similar spores, even when it is performed by qualified and experienced personal.

Beside microscopical analysis, airborne fungal spores are often identified by cultivation of sampled organisms on culture media. However, as with spores, the identification is often limited due to small morphological differences between fungal species. Even worse, there can be large quantitative and qualitative differences between the total number of collected spores and those viable that can be cultured, counted and identified (at least to the genus level) [13]. The main drawback of cultivation is that less than 20% of known fungal species can be grown in culture, and not even on the same medium [14]. Recent method used for analysis and identification of airborne bioparticles is molecular study of DNA sequences obtained directly from environmental samples (metagenomics) [15,16]. The disadvantages of this method are its complexity (equipment and time consuming techniques), high costs, and low ratio of known species represented with at least one DNA sequence (needed for comparison) in public nucleotide sequence databases such as GenBank.

Therefore, aeroallergen-monitoring networks have an urgent need for a rapid, precise and inexpensive method of bioparticle identification. The last decade saw a growing number of experimental works dedicated to identification methods based on vibrational (infrared and Raman) spectra of spores and pollens [17–28]. As opposed to microscopical analysis of bioparticles, vibrational spectroscopy offers a bias-free approach based on chemical characterization via identifiable spectral features. It has been clearly demonstrated that vibrational spectra of spores and pollen can be used for simple and rapid identification through straightforward correlation between spectra and biochemical composition. Moreover, vibrational spectroscopy enables analysis of spores that cannot be grown under laboratory conditions, as well as unviable spores. This is quite important since even unviable spores and spore fragments can cause allergic reaction in susceptible individuals. Out of the two spectroscopic methods, Raman spectroscopy has gained more attention, although due to sample fluorescence and laser-induced degradation the method is limited to light-coloured samples. Since a number of fungal species have dark-coloured spores, evidently IR spectroscopy has more potential for general studies of bioparticles.

The present work shows the application of Fourier transform infrared spectroscopy (FTIR) for analysis and identification of aeroallergen bioparticles. The new measurements reported in this publication cover: 1) the characterization of fungal spores and plant pollens by FTIR, as well as their differentiation, since usually both types of bioparticles are present side by side in a sample during aeroallergenic monitoring; 2) the potential role of FTIR methodology in fungal identification and taxonomy; 3) the estimation of morphological features of pollen and spores based on their FTIR spectra; and 4) the assessment of temporal decay of the biochemical composition of stored fungal spores. The methodology has been tested on Basidiomycota fungal spores that have been found to be the predominant airborne bioparticles [12,29]. Along with fresh basidiospores, the study has been conducted on the archived samples collected within the last 50 years. Regarding pollen, the measured samples cover the leading aeroallergen plants: grasses and sedges, angiosperm trees from birch and beech families, and conifers from pine and cypress families.

## Materials and Methods

### Samples

The preliminary study of fungal spores comprised FTIR spectra measurements of seven samples of archived spores belonging to seven species: *Boletus depilatus* and *Leccinum pseudoscabrum* (family Boletaceae, order Boletales), *Agrocybe pediades* (family Strophariaceae, order Agaricales), *Amanita citrina* and *A*. *pachyvolvata* (family Amanitaceae, order Agaricales), and *Geastrum triplex* and *G*. *fimbriatum* (family Geastraceae, order Geastrales). The samples were collected between 1999 and 2006 at various locations in Croatia. For the principal study of fungi, fresh samples of Basidiomycota spores were collected between August and October 2010 at various locations within Zagreb County and Grad Zagreb County, Croatia. In addition to fresh samples, the study comprised measurements of archived basidiospores, stored under r.t. (room temperature), that were collected between 1961 and 2007 at various locations in Europe. Altogether, 64 spore samples from members of class Agaricomycetes were measured, belonging to three genera represented with two species per genus: *Lycoperdon* (11 samples of *L*. *perlatum* and nine samples of *L*. *pyriforme*; family Agaricaceae, order Agaricales), *Scleroderma* (nine samples of *S*. *areolatum* and five samples of *S*. *citrinum*; family Sclerodermataceae, order Boletales), and *Geastrum* (16 samples of *G*. *triplex* and 14 samples of *G*. *fimbriatum*; family Geastraceae, order Geastrales) (Table A in S1 File). Fungal spores from the samples of the genera *Geastrum*, *Lycoperdon*, and *Scleroderma* were obtained directly from their fruitbodies which were shaken to release the spores on the glass slides. Spores of all other fungal species were obtained from their spore prints which were scraped. All samples were stored at r.t.

Samples of pollen were collected between March and June 2011 at the Botanical Garden of the Faculty of Science, the University of Zagreb, Grad Zagreb County, Croatia. In total 121 samples were collected, each of them belonging to different species of Poales, Fagales and Pinales plant orders: 14 species belonging to four genera of Cyperaceae (sedge) family, 28 species belonging to 15 genera of Poaceae (grass) family, 13 species belonging to two genera of Fagaceae (beech) family, 13 species belonging to five genera of Betulaceae (birch) family, 24 species belonging to 13 genera of Cupressaceae (cypress) family, and 29 species belonging to five genera of Pinaceae (pine) family (Table B in S1 File). The pollen samples were collected directly from plants at flowering time. The samples were kept at r.t., and in general their IR spectra were recorded within 48 h.

The fresh samples were obtained through fieldwork in the Republic of Croatia. The study is a part of government-funded research, and has been conducted with the full cooperation of the The Ministry of Education, Sciences and Sports of the Republic of Croatia, and the administrations of the involved institutions: the Ruđer Bošković Institute and the University of Zagreb. The field studies did not involve endangered or protected species.

For identification of basic biochemicals in spores and pollen numerous spectra of model compounds were measured, including lipids, carbohydrates and proteins. Model compounds were purchased from Merck (Darmstadt, Germany) and Sigma-Aldrich (St. Louis, United States), and used without further purification. The model compounds included tristearin (1,3-di(octadecanoyloxy)propan-2-yl octadecanoate), triolein (2,3-bis[[(Z)-octadec-9-enoyl]oxy]propyl (Z)-octadec-9-enoate), phosphatidistearoylcholine (1,2-distearoyl-rac-glycero-3-phosphocholine), phosphatidioleylcholine (1,2-dioleoyl-sn-glycero-3-phosphocholine), stearic acid (octadecanoic acid), oleic acid ((9Z)-octadec-9-enoic acid), cellulose, amylose, amylopectin, arabinoxylan, pectin, sucrose, trehalose, fructose, glucose, chondroitin sulfate C, β(1,3)D-glucan, chitosan, chitin, gluten, α-casein, β-casein, κ-casein

### Microscopy

Photographs of pollen and spores were obtained by a digital camera Olympus Colorview IIIu attached on the Olympus BX51 light microscope, after being mounted in 2.5% potassium hydroxide (KOH) solution. Pollen grains were photographed through a UPlanFl 40× objective, and spores through a UPlanSApo 100× oil objective. Pollen grains of *Abies cephalonica* and spores of *Geastrum triplex*, *Scleroderma areolatum*, and *S*. *citrinum* are shown with composite extended depth of field images made from stack of images by CombineZP software.

### Infrared measurements

Reflectance IR spectra were recorded with a resolution of 4 cm^-1^ using cosine apodization on an ABB Bomem (Quebec City, Canada) MB102 single-beam spectrometer, equipped with CsI optics, DTGS detector and the single-reflection attenuated total reflectance (SR-ATR) accessory. The ATR IR spectra were recorded by measuring approx. 0.5 mg of a sample, with a total of 30 scans using the horizontal SR-ATR diamond prism with 45° angle of incidence on a Specac (Slough, United Kingdom) Golden Gate ATR Mk II or a Specac High Temperature Golden Gate ATR Mk II. Each spectrum was recorded as the ratio of the sample spectrum to the spectrum of the empty ATR plate. Spectra of crystal lipids and carbohydrates were recorded above their melting temperature, and again at r.t. after cooling to obtain a spectrum of the amorphous phase (liquid and/or glass phase).

### Spectral pre-processing and data analysis

The FTIR spectra were pre-processed prior to calibration: all spectra were smoothed or transformed to second derivative spectra by the Savitzky–Golay algorithm using a polynomial of degree two and a window size of 15 points in total, followed by normalization by multiplicative signal correction (MSC) or extended multiplicative signal correction (EMSC), an MSC model extended by a linear and quadratic component) [30–32]. The spectral region of 800 cm^-1^ to 1900 cm^-1^ was selected for data analysis. In the EMSC pre-processing of non-derivated spectra, the spectral region of chemical absorbance was down-weighted, and region devoid of any chemical absorbance was up-weighted, by applying a weighting vector that ensured a stable baseline in all spectra (vector value 1 in the whole spectral region except the region 1770–1900 cm^-1^, where the weighting vector was set to 10). Pre-processed spectra were used to evaluate biochemical similarities between pollen samples by using principal component analysis (PCA) and hierarchical cluster analysis (HCA). HCA plots (dendrograms) were calculated with Euclidian distance measure and Ward distance clustering algorithm.

The spectral pre-processing and data analyses were performed by algorithms developed in-house in the environment of Matlab, R2014a (The Mathworks Inc., Natick, United States).

## Results and Discussion

### Sample set

The study has covered three plant orders that are highly represented in aerobiological studies: Fagales, Poales and Pinales. Moreover, the measured pollen genera are responsible for the majority of pollen allergies in the Northern hemisphere during late winter to early summer period, most noticeably *Betula* and *Alnus* (Betulaceae), *Quercus* (Fagaceae), *Cupressus* and *Juniperus* (Cupressaceae), *Poa* and *Festuca* (Poaceae) and *Carex* (Cyperaceae). Although Pinaceae pollen allergy is relatively uncommon and clinically insignificant, Pinaceae samples have been incorporated into the study due to their high occurrence in aerobiological monitoring.

Regarding fungal spores, the FTIR measurements have covered basidiospores. Basidiospores are sexual propagative cells of Basidiomycota produced after meiosis on spore-producing structures called basidia. Although all the samples belong to only one fungal phylum, they offer a great trial set for testing the FTIR methodology. Recent studies have shown that Basidiomycota are often the predominant airborne fungal spores [12,29,33]. The allergenicity of fungal spores has not been studied as much as that of pollen, and most of the studies have focused on asexually produced mitospores, e.g. in *Alternaria*, *Cladosporium* and *Aspergillus* genera. However, it was shown recently that sensitization to basidiospores can be more widespread than sensitization to mitospores [34,35]. The presented study has covered allergenic fungal genera that have wide geographical distribution and ability to release numerous spores. Since spores of these species are easily obtainable from dry fruiting bodies, the study has achieved diversity of adequate samples of different ages and from various geographic locations. Secondly, spores of Basidiomycota are difficult to identify since most basidiospores are small and problematic to characterize under an optical microscope, and in addition, the majority will not grow on laboratory media. As a result, aerobiological studies are often limited to differentiation of less than 20 basidiospore categories, where each category can cover several fungal families [35]. Thirdly, roughly half of the Basidiomycota produce dark-coloured spores that cannot be measured by Raman spectroscopy due to strong fluorescence background and laser-induced degradation. The preliminary study has covered a range of Basidiomycota with different coloured spores, from white to dark brown, and all of them were successfully measured. To test the advantage of FTIR spectroscopy over Raman spectroscopy, the principal study has covered only dark-coloured spores.

The principal FTIR measurements of basidiospores have been conducted on six species of Agaricomycetes. Even though the measured fungal species belong to three different orders of Agaricomycetes, they have common spore propagation strategy that is noticeable by the same type of fruiting body—gasterocarp. Unlike most Basidiomycota, where spores are produced on the surface of a fruiting body and are actively discharged in the air (ballistospores), spores in gasterocarp are produced internally and discharged after an external impact (statismospores). Due to peculiar-looking spheroidal fruiting bodies and distinctive discharge of spores this group of fungi is often referred to as puffballs. The two measured genera of puffballs, *Scleroderma* and *Geastrum*, are known allergenic fungi [36].

### Morphology of fungal spores and pollen

**Fig 1:**
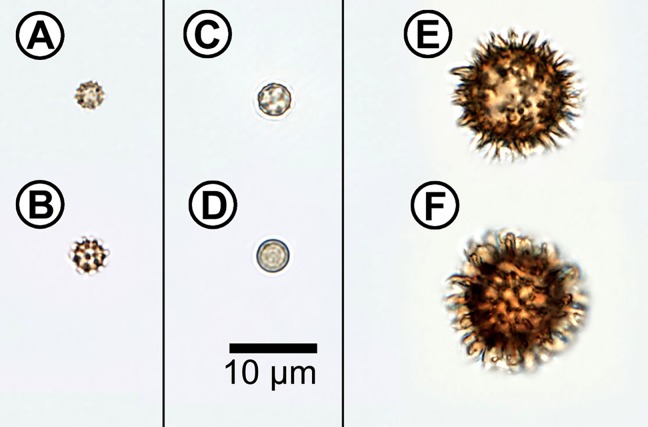
Microscope image of fungal spores. (**A**) *Geastrum fimbriatum*; (**B**) *Geastrum triplex*; (**C**) *Lycoperdon perlatum*; (**D**) *Lycoperdon pyriforme*; (**E**) *Scleroderma areolatum*; (**F**) *Scleroderma citrinum*.

**Fig 2:**
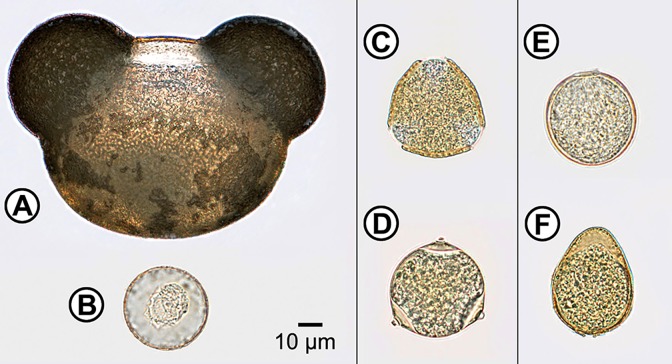
Microscope image of pollens. (**A**) *Abies cephalonica* (Pinaceae), equatorial view; (**B**) *Cupressus sempervirens* (Cupressaceae), polar view; (**C**) *Quercus robur* (Fagaceae), polar view; (**D**) *Carpinus betulus* (Betulaceae), polar view; (**E**) *Bromus erectus* (Poaceae), equatorial view; (**F**) *Carex pendula* (Cyperaceae), equatorial view.

Assorted samples of spores and pollen were observed using light microscope under standard magnifications (40× and 100× objectives) for morphological characterization of spores and pollen (Figs 1 and 2). The samples show various and taxon-specific morphological features that are well described in mycological and palynological literature, therefore only the most prominent characteristics are stated.

The measured basidiospores are smaller than pollen samples with diameters ranging from 3 to 19 μm. *Geastrum* spores are spherical and verrucose, with diameters (including ornament) of 3–4.5 μm and 4–6 μm for in *G*. *fimbriatum* and *G*. *triplex*, respectively (Fig 1A and 1B). The spores are brown in mass, with warts of a length up to 0.5 μm and up to 0.8 μm for *G*. *triplex* and *G*. *fimbriatum*, respectively. *Lycoperdon* spores are spherical, with sizes that range from 3 to 5.5 μm in diameter. *L*. *perlatum* spores are finely verrucous and are olive- to grey-brown in mass, while *L*. *pyriforme* spores are almost smooth and olive-brown in mass (Fig 1C and 1D). *Scleroderma* spores are the largest of the three analysed genera, with diameters ranging from 10 to 19 μm (including ornament), and both species have spherical dark-brown spores. *S*. *areolatum* spores have a diameter of 11–19 μm, and their surface is covered with large pointed spines up to 3 μm high (Fig 1E). Finally, *S*. *citrinum* spores typically have a diameter in the range of 10–16 μm, and their surface shows an up to 2.5 μm high ornament forming an incomplete reticulum (Fig 1F).

The diameter of measured pollen grains varied from approximately 15 μm to more than 100 μm. Conifer (Pinales order) grains show the largest variations in size and shape. Pinaceae grains are yellow, with diameters ranging from 75 to 130 μm, and have large sporopolleninous hollow projections (saccus) from the central body of the pollen grain. Most Pinaceae grains have two sacci, which can be easily depicted in the equatorial view (Fig 2A). Cupressaceae pollen grains have a uniform spheroidal and monoporate morphology with a specific sporopolleninous ornamentation called Ubisch bodies (Fig 2B). They have much smaller sizes than Pinaceae pollen, with diameters varying between 15 to 35 μm, and their colours range from yellow to light brown and red-brown.

The pollen grains of angiosperms (Fagales and Poales orders) measured in this study are yellow, and their size is typically varying between 25 to 50 μm. Fagaceae pollen are spheroidal to prolate, with three elongated apertures (Fig 2C). Betulaceae grains are spheroidal to oblate, with three or more apertures situated at the equator, and with genus-specific thickening of the pollen wall (Fig 2D). Poaceae grains have uniform spheroidal and monoporate morphology, with a distinct thickening of the pollen wall called annulus surrounding a porus (Fig 2E). Cyperaceae grains are oblate spheroidal to suboblate (wedge-shaped), inaperturate to polyaperturate, and with an exine structure called operculum covering an aperture (Fig 2F).

### Spectroscopic characterization and differentiation of fungal spores and pollen

FTIR measurements of spores employing an SR-ATR accessory have been performed on basidiospore samples of various taxa, with sample colour ranging from white to dark brown. All samples were measured successfully regardless of spore colour, which has not been the case for the reported Raman studies [20,25]. FTIR spectra of different pollen samples can be easily obtained by the same technique, as shown in our previous studies [24,27].

**Fig 3:**
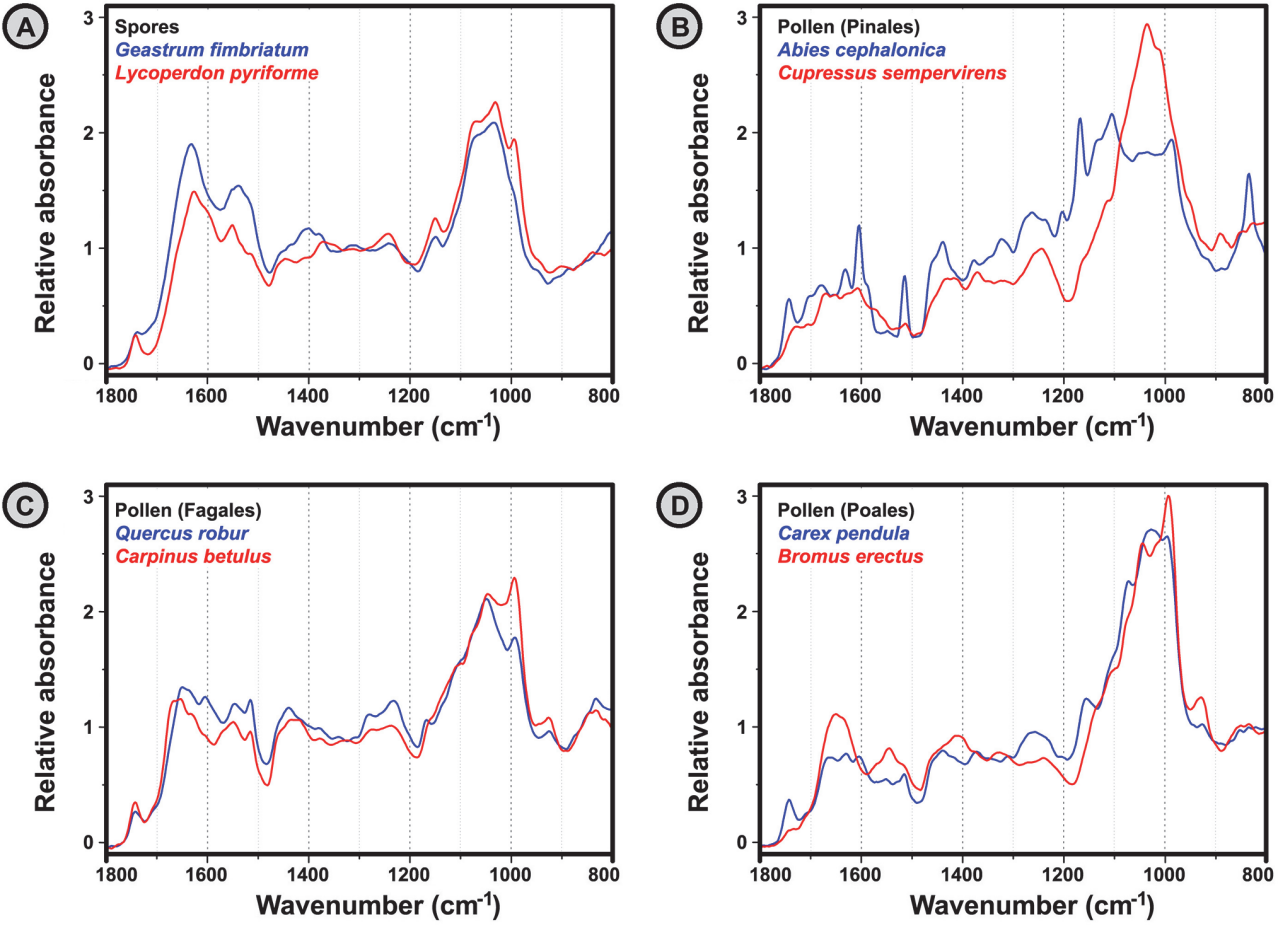
FTIR spectra of representative samples of spores and pollen. (**A**) spores of *Geastrum fimbriatum* and *Lycoperdon pyriforme*; (**B**) Pinales pollen of *Abies cephalonica* (Pinaceae) and *Cupressus sempervirens* (Cupressaceae); (**C**) Fagales pollen of *Quercus robur* (Fagaceae) and *Carpinus betulus* (Betulaceae); (**D**) Poales pollen of *Bromus erectus* (Poaceae) and *Carex pendula* (Cyperaceae). The spectral set was normalized by MSC (with weighting vector).

**Fig 4:**
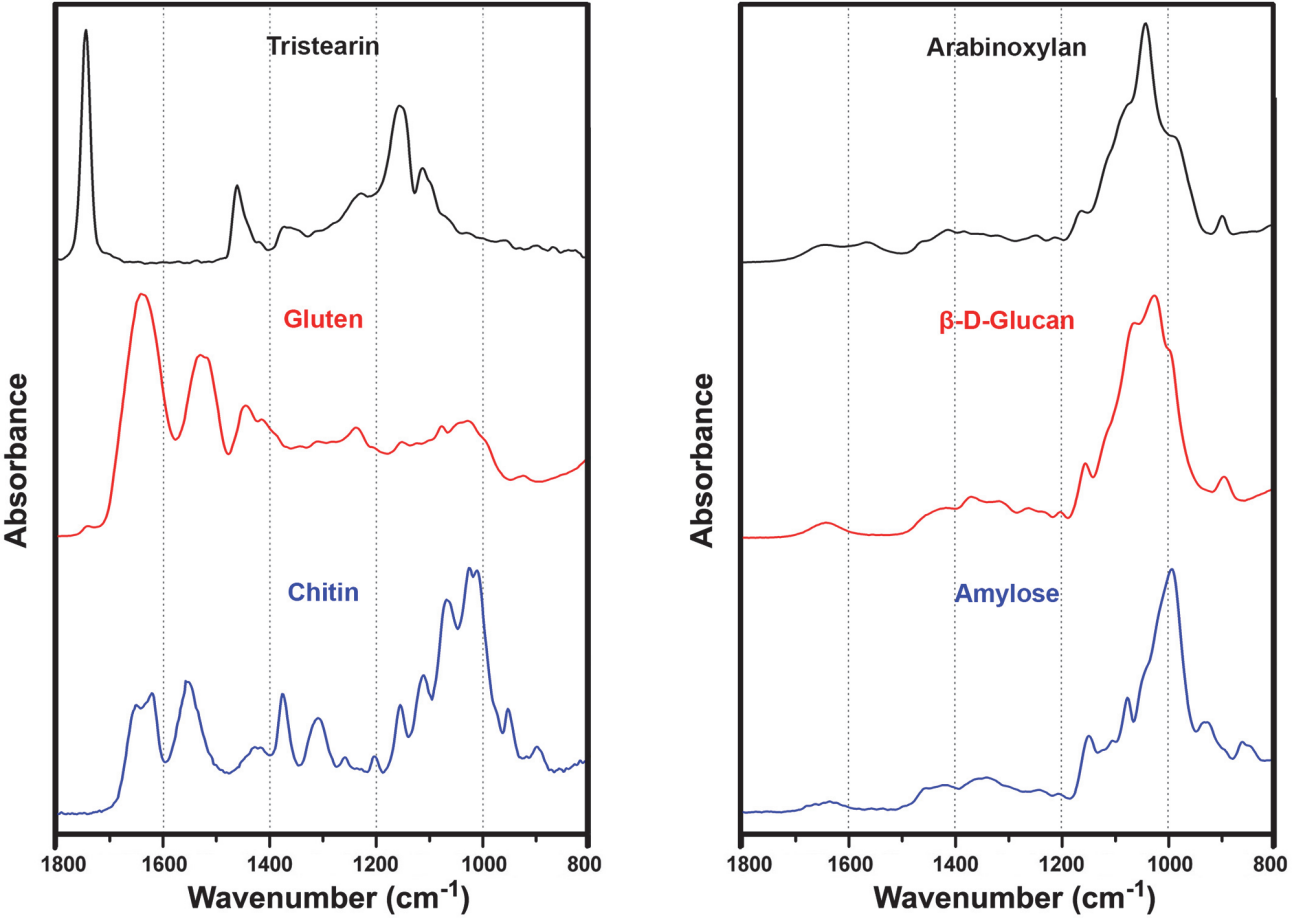
Infrared spectra of biochemicals. FTIR spectra of lipid (tristearin), protein (gluten) and polysaccharides (chitin, arabinoxylan, β-D-glucan, amylose); for better viewing the spectra are offset.

The analyses of IR spectra belonging to pollen and spores show that these bioparticles have very distinct spectral features (Fig 3). An infrared spectrum of a bioparticle can be divided into specific regions containing signatures of lipids, proteins, carbohydrates, and grain wall biopolymers such as sporopollenin and chitin. The IR spectra of basic biochemicals are presented in Fig 4, with depicted main characteristic vibrational bands. Since these chemicals are the main components of bioparticles, either as structural chemicals or as nutrients, they are responsible for the majority of phenotypic attributes. The corresponding spectral signals of these chemicals are highly specific, and thus FTIR spectroscopy is excellent tool for biochemical analysis of plants and fungi. For example, fungal spores have quite high absorption in the short wavelength spectral region (1700–1500 cm^-1^) due to chitin in the cell wall, as well as high content of proteins (Fig 3A). Both type of compounds (Fig 4, gluten and chitin) are characterized by two strong and broad bands, at 1650 cm^-1^ (amide I: C = O stretch) and 1550 cm^-1^ (amide II: NH deformation and C–N stretch). On the other hand, the pollen grains of *Cupressaceae* species, such as *Cupressus sempervirens* (Fig 3B), have high absorption in the long wavelength spectral region (1200–900 cm^-1^) due to high content of carbohydrates, particularly cellulose in the grain wall. The vibrational bands associated with carbohydrates, such as C–O–C and C–OH stretch, are predominant in this spectral region (Fig 4, arabinoxylan, β-D-glucan and amylose). Finally, the spectral difference between pollens belonging to Poaceae (grasses) and Cyperaceae (sedges) families is largely based on trygliceride content. Triglycerides (Fig 4, tristearin) are characterized by the strong vibrational band at 1745 cm^-1^ (C = O stretch), and the higher absorption at 1745 cm^-1^ in the spectrum of *Carex pendula*, when compared with the spectrum of *Bromus erectus*, can be clearly seen (Fig 3D). It should be noticed that the so-called coding and functional biochemicals, such as nucleic acids and enzymes, constitute minor features of an IR spectrum since they are not present in significant amount. As a result, FTIR measurement of pollen and spores offers complementary information that is overlooked by genomic and proteomic methods.

**Fig 5:**
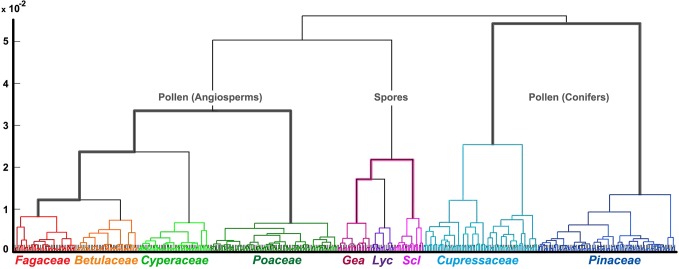
Cluster analysis of spectra belonging to fresh pollen and spores. HCA dendrogram of IR spectral data set of fresh pollen and spores (three spectra per sample; second derivative and MSC corrected spectra) with depiction of plant families and fungal genera (Gea, *Geastrum*; Lyc, *Lycoperdon*; Scl, *Scleroderma*).

In Fig 5 A HCA dendrogram of IR spectra of fresh pollen and spores is shown including in total 121 pollen samples and 18 spore samples. The HCA (Fig 5), as well as the PCA conducted on the same sample set (Fig. A in S1 File), show that the spectra of basidiospores are well separated from the pollen spectra of major aeroallergenic plant orders: Fagales, Poales and Pinales. Moreover, IR spectra of samples belonging to distinct plant families (Fagaceae, Betulaceae, Cyperaceae, Poaceae, Pinaceae and Cupressaceae), as well as fungal genera (*Lycoperdon*, *Scleroderma*, *Geastrum*), are well separated from each other.

**Fig 6:**
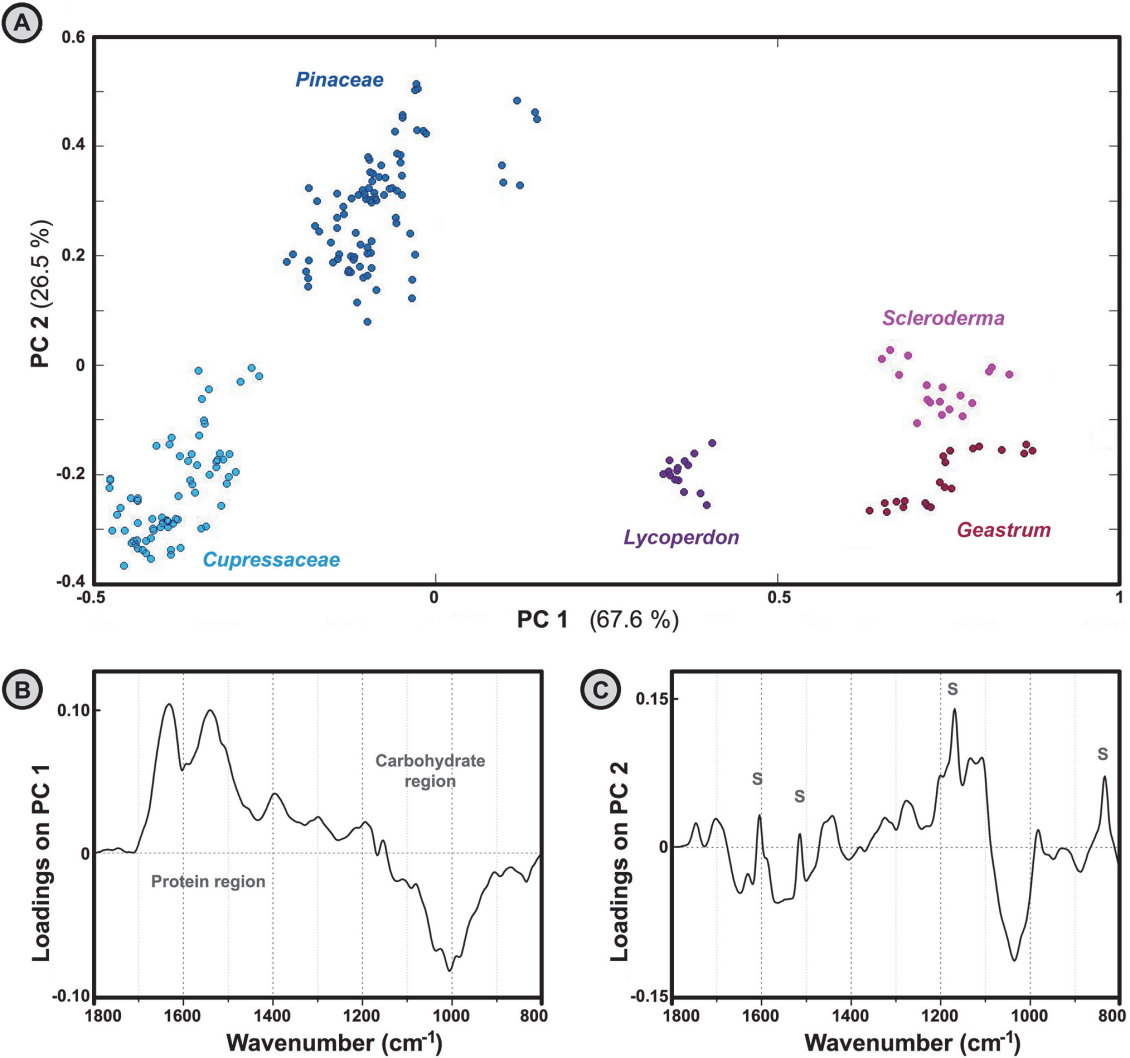
Analysis of spectra belonging to fresh spores and Pinales pollen. PCA plot of IR spectral data set of fresh spores and Pinales pollen (three spectra per sample; MSC corrected spectra), with depiction of plant families and fungal genera. The percent variances for the first five PCs are 67.57, 26.50, 1.77, 1.26 and 0.65. (B) Loadings plot on the first two principal component of the PCA; the marked signals (S) are associated with vibrational bands of sporopollenins.

PCA analysis of Agaricomycetes spores and Pinales (conifers) pollen shows substantial differences in the biochemical composition between these two groups of organisms (Fig 6A). Based on the examination of the first principal component (Fig 6B), the predominant difference between spores and Pinales pollen is in protein-to-carbohydrate ratio. As mentioned previously, the IR spectra of Pinales pollen show dominant absorption due to vibrational bands in the ‘carbohydrate region’ (1200–900 cm^-1^). On the other hand, spores show strong IR absorption of amide I and amide II bands in the ‘protein region’ (1700–1500 cm^-1^). As can be seen in Fig 6B, PC1 has high negative and high positive loadings in the ‘carbohydrate region’ and the ‘protein region’ respectively. Therefore, PC1 separation is based on the predominantly amide-based chemical composition of spores (high positive PC1 values), and predominantly carbohydrate-based chemical composition of Pinales pollen (high negative PC1 values). Moreover, IR spectra of spores are well separated from the spectra of Pinaceae (pine) family pollen based on the second principal component as well (Fig 6A). Pinaceae spectra have characteristic bands at 1605, 1515, 1169 and 833 cm^-1^ (Fig 3B). These specific absorptions are attributed to sporopollenins, the highly resistant and complex biopolymers that constitute the outer layer of pollen grain wall (exine). As can be seen in Fig 6C, PC2 has high positive loadings for these sporopollenins bands, resulting with high positive PC2 values for all Pinaceae samples (Fig 6B). In general, the chemical composition of Pinaceae pollens and fungal spores is extremely different, resulting with clear discrimination of their IR spectra.

**Fig 7:**
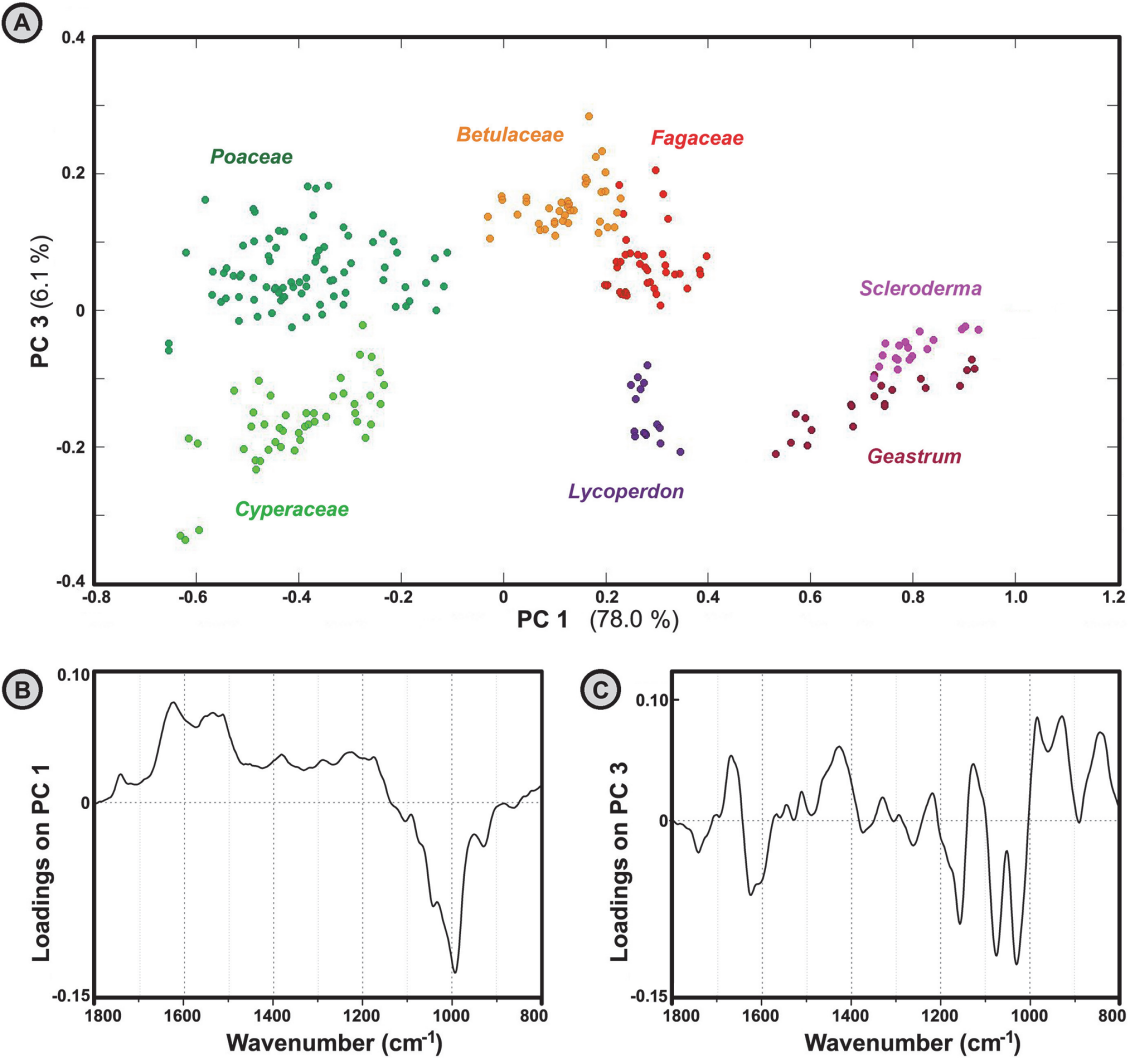
Analysis of spectra belonging to fresh spores and Fagales pollen. PCA plot of IR spectral data set of fresh spores and Poales and Fagales pollen (three spectra per sample; MSC corrected spectra), with depiction of plant families and fungal genera. The percent variances for the first five PCs are 78.00, 9.95, 6.07, 1.69 and 0.97. (B) Loadings plot on the first two principal component of the PCA.

The differences between IR spectra belonging to basidiospores and angiosperm pollen (Poales and Fagales plant orders) are extensive, although to a lesser degree than is the case with conifer pollen (Figs 3 and 7). Analogous to the previous case, the predominant difference between spores and angiosperm pollen is in the protein-to-carbohydrate ratio, as shown by loadings plot on the first principal component of the PCA (Fig 7B). Based on the examination of the principal components, the differences between spores and Fagales pollen can be tentatively assigned to differences due to carbohydrate composition, as represented by variations of the vibrational bands in the carbohydrate region (Fig 7C). The second principal component explains spectral differences within Basidiomycota and within Poales clades, and does not show differences between spores and pollen (results not shown).

**Fig 8:**
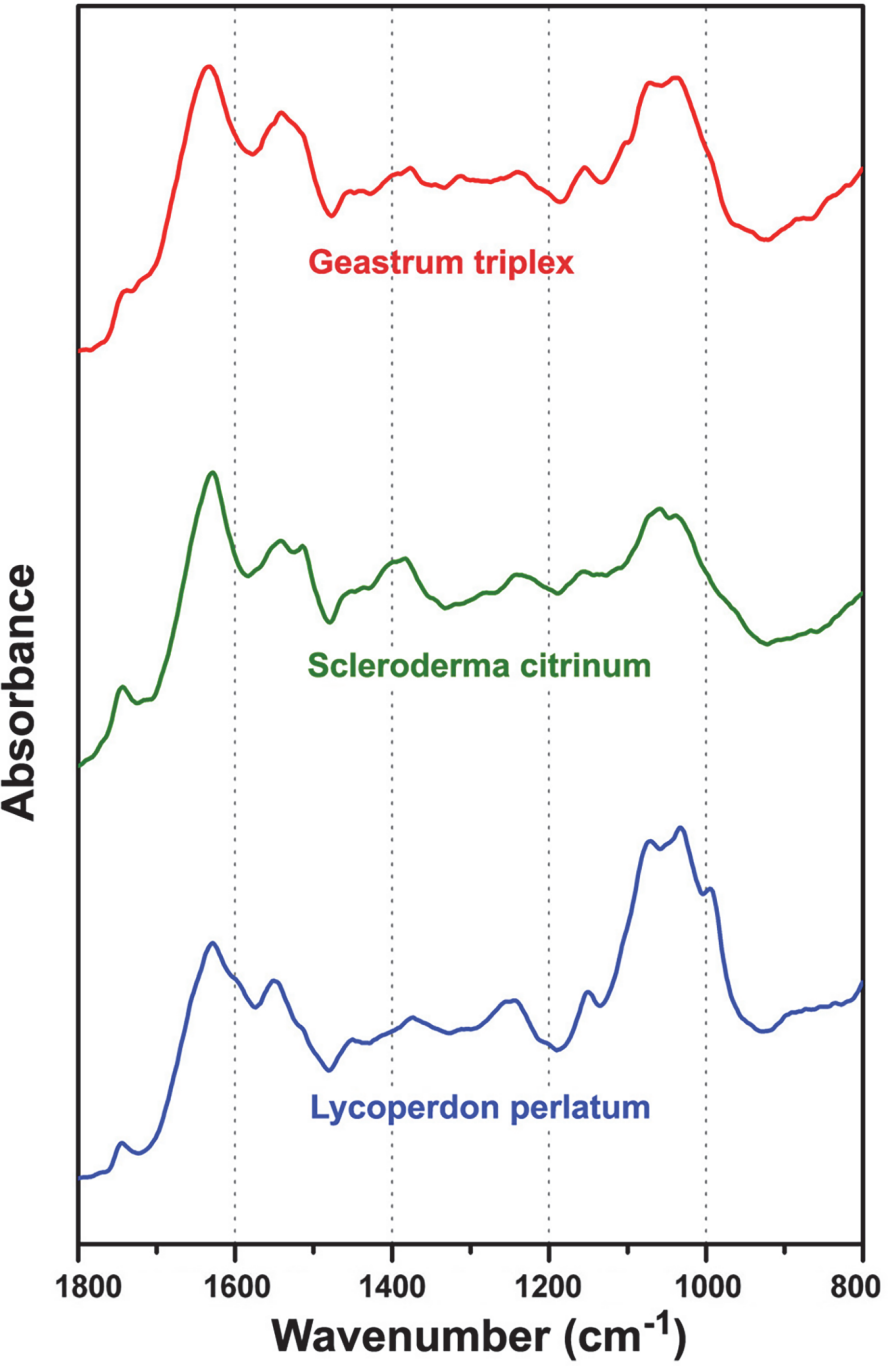
Infrared spectra of fungal spores. FTIR spectra of representative samples of *Geastrum triplex*, *Lycoperdon perlatum* and *Scleroderma citrinum* spores. For better viewing the spectra are offset.

**Fig 9:**
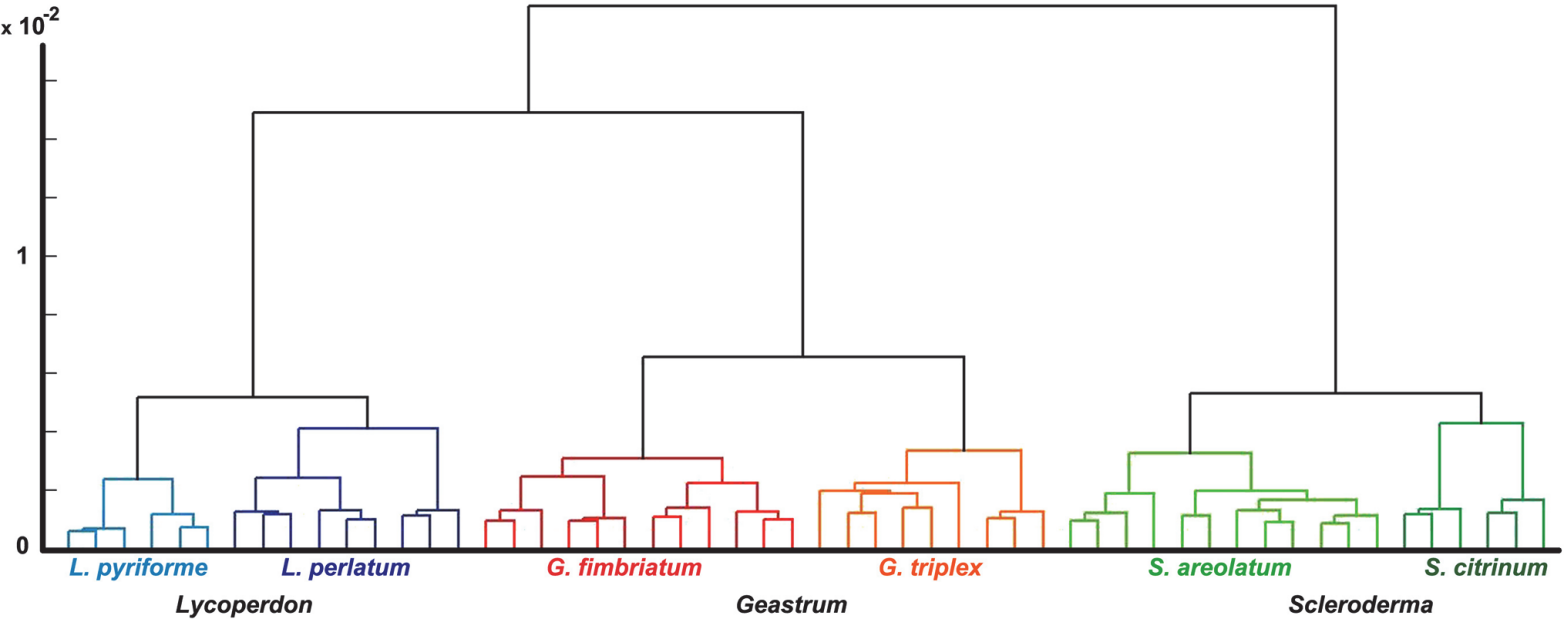
Cluster analysis of spectra belonging to fresh spores. HCA dendrogram of IR spectral data set of fresh spores (three spectra per sample; second derivative and EMSC corrected spectra), with depiction of fungal genera and species.

**Fig 10:**
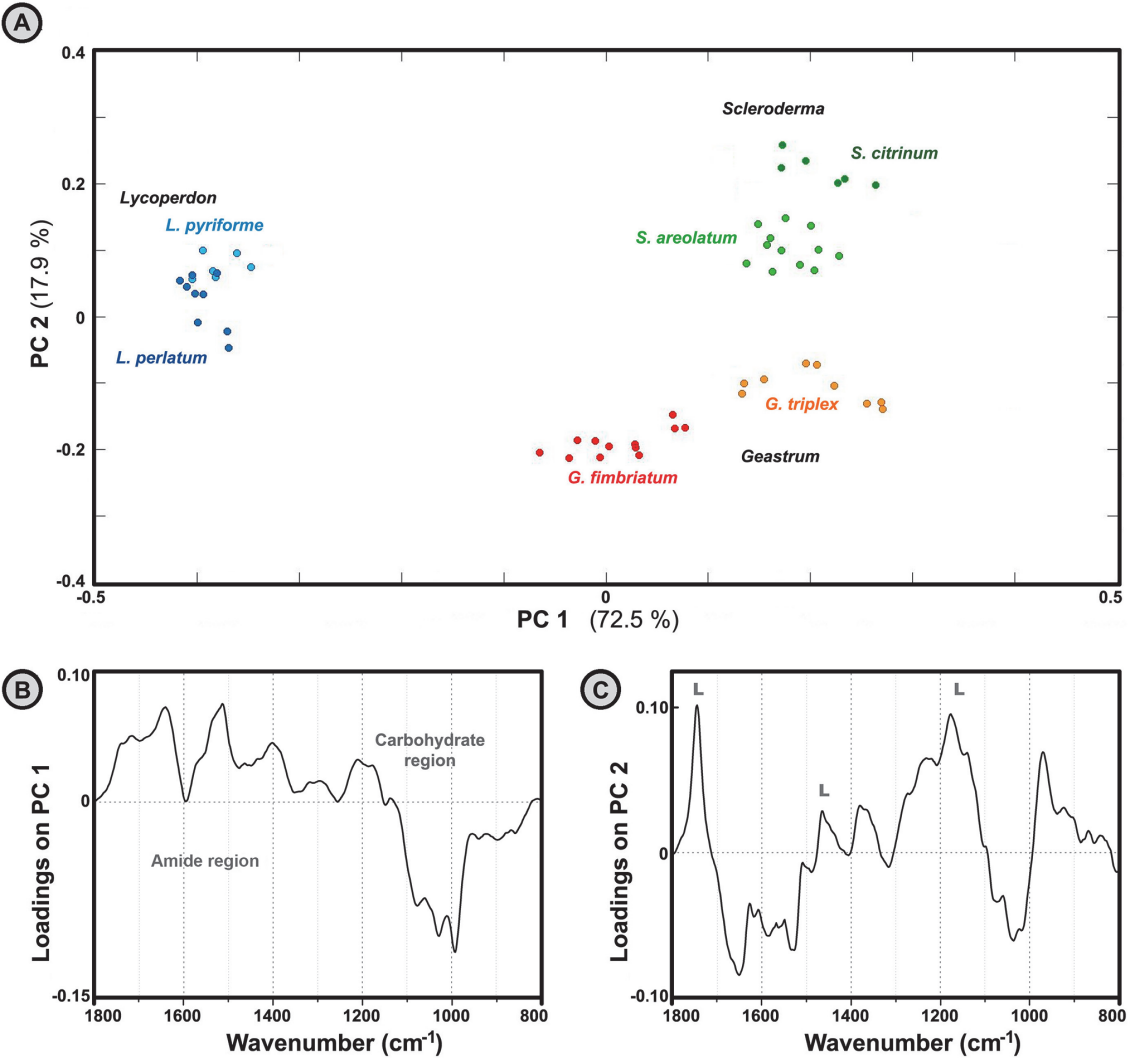
Analysis of spectra belonging to fresh spores. PCA plot of IR spectral data set of fresh spores (three spectra per sample; MSC corrected spectra), with depiction of fungal genera and species. The percent variances for the first five PCs are 72.53, 17.94, 3.69, 2.56 and 1.16. (B) Loadings plot on the first two principal component of the PCA; the marked signals (L) are associated with vibrational bands of lipids.

The IR spectra of fresh spore samples (Fig 8) have sufficient variations for differentiation of genera and species (Figs 9 and 10A). Fig 10B shows high positive loadings on the PC1 for amide bands that can be attributed to chitin and proteins (1700–1500 cm^-1^ region), as well as high negative values for carbohydrate bands (1200–900 cm^-1^ region). Therefore, it can be assumed that the main differences in chemical composition are based on chitin-to-β-glucan ratio. Among the three measured genera, *Lycoperdon* spores have the most negative PC1 values, and thus presumably the lowest chitin-to-β-glucan ratio. Additional spectral differences probably arise due to variations in lipids, as indicated by high positive loadings on PC2 associated with lipid bands (Fig 10C).

### Infrared spectra and morphology of bioparticles

Alongside absorption signals of chemical constituents, IR spectrum of a sample always includes interferent signals due to nonchemical effects, such as light scattering due to sample morphology and changes in the refractive index. These effects cause unwanted phenomena in IR spectra in the form of various baseline and background features [30–32]. Since interferent signals can hinder chemical interpretation of the spectra they are often corrected by pre-processing methods. One approach includes transformation of recorded data, such as calculating derivatives or spectral smoothing, both of which can be obtained by applying the Savitzky–Golay (SG) algorithm. Another approach includes scatter-corrective pre-processing methods, such as MSC and EMSC. EMSC is a model-based pre-processing method that allows explicit parameterization of physical interferent information, thus enabling the separation of physical and chemical information from the spectral data [30]. Both the SG and the EMSC procedures are often used cooperatively to more effectively reduce unwanted phenomena in IR spectra. In the analysed data sets, the IR spectra were transformed into second derivatives to emphasize bandwidths, positions, and separations. This has resulted in a good separation of various taxonomic groups, as shown by cluster analyses (Figs 5 and 9). However, since original (non-derivated) data offer more straightforward comparison with the spectra of biochemicals and thus simpler chemical interpretation, the original spectral form was preferred in the PCA analyses presented in Figs 6, 7 and 10.

Although baseline, background and other anomalous features are often treated as unwanted in spectral analysis, they can hold important information for sample identification [30]. This is particularly true in the case of pollen and spores where anomalous features are closely related to a characteristic morphology of a bioparticle. For example, light scattering is directly related to bioparticle morphology, which varies substantially in shape, size, and texture across plant and fungal taxa. In addition, scattering effects are dependent on the refractive index of the bioparticle and refractive index variations within a bioparticle, due to variation in chemical composition between different substructures (such as grain wall layers in pollen and spore). This property varies substantially across plant and fungal taxa as well as morphology. Therefore, anomalous features could be closely correlated with phylogenetic groups and could serve for identification of bioparticles, alongside chemical information.

**Fig 11:**
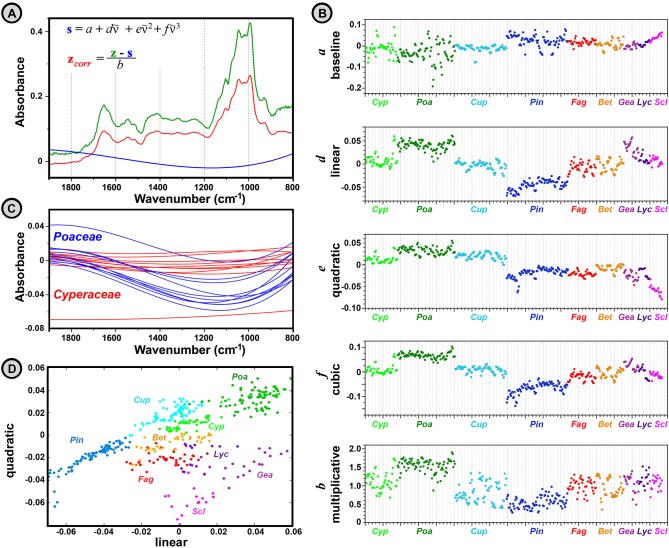
Separation of physical and chemical information in spectra of pollen and spores. (**A**) FTIR spectra of *Poa pratensis*: recorded spectrum (green), scatter estimate with constant, linear, quadratic, and cubic terms (blue), and EMSC corrected spectrum (red) (multiplicative parameter *b* for *Poa pratensis* is 1.65); Savitzky–Golay smoothing (quadratic polynomial with window size 11) was obtained prior to EMSC correction with weighting vector. (**B**) EMSC parameters, and (**C**) scatter estimates for 10 *Poaceae* (blue) and 10 *Cyperaceae* spectra (red). (**D**) plot for linear and quadratic EMSC parameters for measured samples: Cyperaceae (Cyp), Poaceae (Poa), Cupressaceae (Cup), Pinaceae (Pin), Fagaceae (Fag), Betulaceae (Bet), *Geastrum* (Gea), *Lycoperdon* (Lyc), and *Scleroderma* (Scl).

Fig 11A illustrates the separation of physical and chemical information in spectra of pollen and spores. The separation was obtained by an EMSC model with constant, linear, quadratic, and cubic parameter spectra, while, as explained in the Materials and Methods section, the regions with strong variations in chemical absorption were down-weighted. The EMSC separates the recorded spectrum (green spectrum in Fig 11A) into a “corrected chemical spectrum” including chemical absorption bands (red spectrum in Fig 11A), and physical information such as the “baseline” (blue spectrum in Fig 11A), and the effective optical path length (multiplicative parameter *b* in Fig 11A). In the employed EMSC pre-processing, the “baseline” was estimated by the following EMSC parameters: constant, linear, quadratic, and qubic (parameters *a*, *d*, *e*, and *f*, respectively, in Fig 11A). These parameters are used to estimate non-chemical absorption, reflection and scattering of light that is usually caused by irregularities in the propagation medium (i.e. pollen or spore sample). The graphical representation of EMSC parameters (Fig 11B, 11C and 11D) clearly shows that their values are not random but rather specific for taxonomic groups. For example, it is clearly visible from Fig 11C that grass and sedge families have completely different spectral scatter estimates, and the same applies for cypress and pine families as well (Fig 11D). This is not surprising, since we expect that a number of factors connected with composition and morphology of a bioparticle have impact on the resulting ATR FTIR spectrum, even when chemical absorption is disregarded. For instance, a number of parameters depend on physical and chemical properties of a bioparticle: 1) refractive indices of sample substructures, 2) quality of the sample contact with ATR crystal, 3) depth of penetration, and 4) effective path length. This means that the presented EMSC parameters can be used for identification and characterization of pollen and spores alongside chemical absorption spectrum.

Therefore, alongside measurement of chemical composition of pollen and spores, the FTIR methodology offers indirect estimation of their morphology. The measured samples show taxon specific morphological features that are basis for identification and quantification of airborne bioparticles in most of contemporary studies. Pollen grains have two-layered grain wall, with cellulose-based inner layer and sporopollenin-based outer layer. The texture and morphology of these layers can be very complex, while the grain diameter can vary from less than 5 μm to more than 200 μm. The size range of basidiospores is between 2 and 55 μm, and, correspondingly to pollen, spore walls can have a variety of thicknesses, structures and ornamentations. The EMSC parameters, obtained by EMSC pre-processing of FTIR spectra, show taxon-specific range of values. In this way, an indirect physical measurement of a bioparticle can be obtained and used alongside chemical information for identification and characterization.

### Changes in spore chemistry with increasing storage time

Fungal spores can endure considerable periods of stress as a result of stasis and desiccation that permit metabolic activity to be suspended [37]. To measure changes in spore chemistry with increasing storage time we have recorded FTIR spectra of basidiospores that were collected within the last 50 years and archived under room temperature conditions.

**Fig 12:**
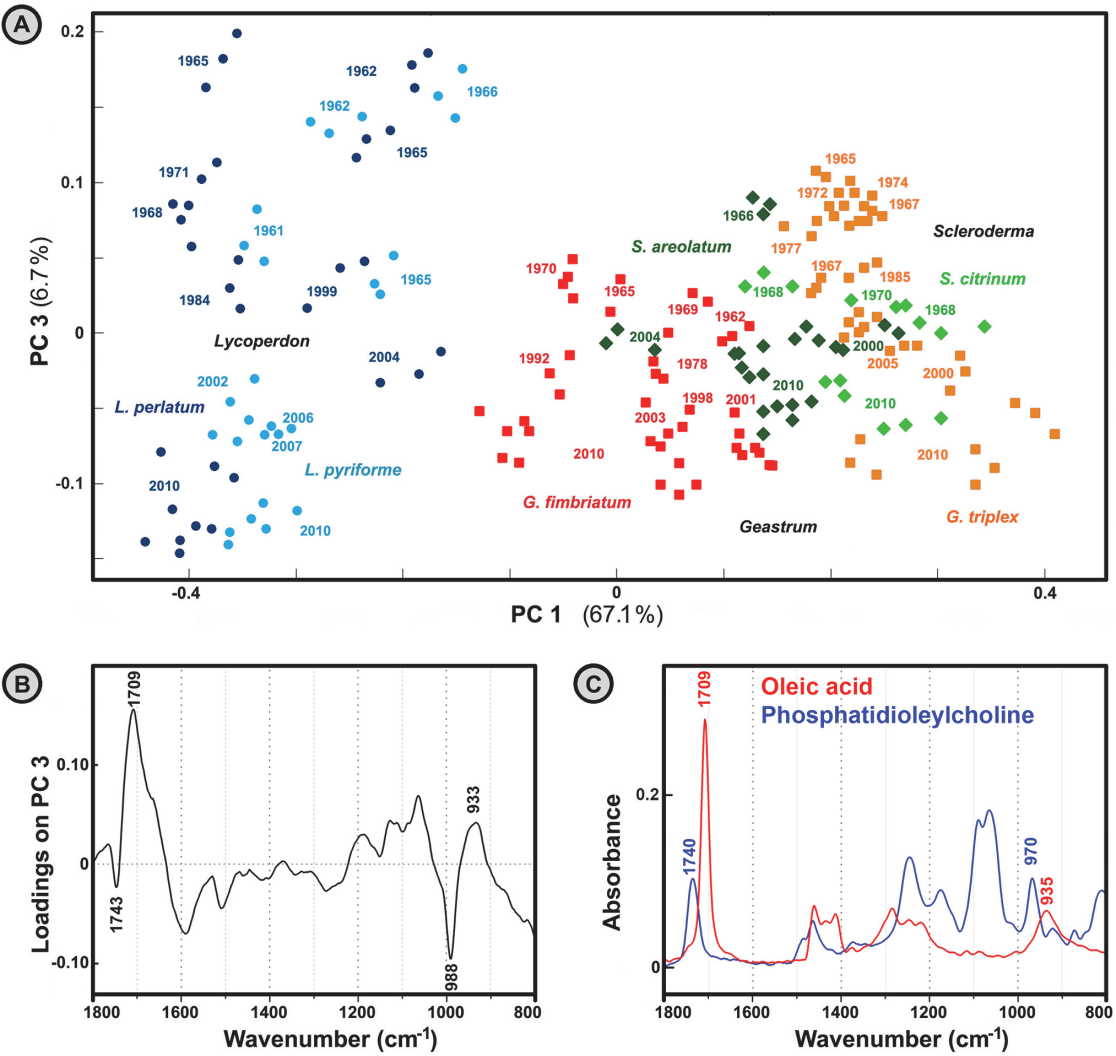
Analysis of spectra belonging to fresh and old spores. PCA plot of IR spectral data set of fresh (2010) and old spores (three spectra per sample; MSC corrected spectra), with depiction of fungal species (● *Lycoperdon*, ■ *Geastrum*, ♦ *Scleroderma*) and year of archiving. The percent variances for the first five PCs are 67.09, 15.22, 6.73, 2.32 and 1.29. (B) Loadings plot on the third principal component of the PCA. (C) IR spectra of oleic acid and phosphatidioleylcholine.

The spectral variations for archived *Geastrum*, *Lycoperdon*, and *Scleroderma* spores are presented in Fig 12. As can be seen, the third principal component is well correlated with chemical changes due to increasing storage time. It should be taken into account that spore samples belong to different fungal populations that have grown under different environmental conditions. Therefore, some of the variation is probably attributed to these genetic and biogeographical differences. Nevertheless, the variation in chemical composition presented in Fig 12 is quite uniform. The PC3 (Fig 12B) has high positive loadings associated with bands at 1709 and 933 cm^-1^ that can be attributed to free fatty acids (C = O stretch in carboxylic acids and C‒O‒H bending, respectively). In addition, it has high negative loadings at 1743 and 988 cm^-1^ that can be attributed to phospholipids (C = O stretch in esters and P‒O‒C antisymmetric stretch, respectively). The declining absorption of phospholipids and the increasing absorption of free fatty acids, with the increasing storage time, could indicate deesterification process in spores due to storage conditions. It is known that phospholipids osmoregulate intracellular material by means of water-enclosing vesicles, while the presence of free fatty acids suggests deesterification of phospholipids via hydrolysis. This process leads to increased phospholipids permeability, followed by leakage of entrapped solutes within vesicles, and finally harmful desiccation of organism that can be lethal. Although the suggested deesterification process is in agreement with the spectral data, it is not fully conclusive whether this is the main process during spore storage, or if other processes are present.

## Conclusions and Outlook

### Method overview

The FTIR method is simple, rapid and economical, since samples can be measured as found in nature without any chemical pre-treatment. In addition, the data acquisition and analysis can be semi-automated, and based on spectral databases. This study, as well as our previous one [27], have demonstrated that a spectral set for such database can be established easily, and it can take into consideration specific biogeographical distribution of species for an area of interest. Therefore, the methodology is independent of specific high-skilled expertise, as opposed to the traditional morphology-based methodology. However, the value of the methodology depends on its ability to discriminate different aeroallergen bioparticles. Our recent studies had established that FTIR spectroscopy of pollen enables detection of phylogenetic variations, such as separation of confamiliar and congeneric species [24,27]. In order to expand the same methodology this study was focused primarily on measurement of fungal spores, with two goals in mind.

The first goal was to establish the spectral features that are characteristic for each group of organisms, and thus enable separation of pollen from spores. As can be seen from the results, the spectral differentiation between pollen and spores is clear-cut. This is the result of different grain wall composition, which is chitin-based in spores, and cellulose- and sporopollenin-based in pollen. Moreover, the sub-differentiation of pollen families is evident as well. Finally, the analysis of the EMSC parameters indicate that the FTIR methodology offers indirect estimation of morphology of pollen and spores. Thus, the study has shown that identification of principal aeroallergen bioparticles can be based on FTIR methodology.

The second goal was to estimate the potential of FTIR spectroscopy for characterization and identification of fungal spores itself. The results show that IR spectra of spores enable sufficient differentiation of genera and species. However, the number of spore samples has been quite small compared to pollen set in our previous studies [24,27], and thus it needs to be expanded in future studies. The analysis of fresh and archived spores shows that chemical composition of spores is preserved quite well even after decades of storage, including the characteristic taxonomy-related signals. The only major chemical components that show signs of significant deterioration are phospholipids, but even this process is not detrimental for identification of archived spores based on characteristic signals. This has a high practical value since the stored spores are a good model for aeroallergen spores that are not in a viable state. In our previous study we have demonstrated that a spectral component of a biochemical class (for instance triglycerides) can be extracted from the data set in order to create partial biochemical classifier [27]. Therefore, the same process can be applied to spore spectra in order to remove the effect of degradation from the spectral data, and hence enable identification of archived or unviable specimens.

The species-specific spectral features are a precondition for applying FTIR in monitoring of aeroallergens. However, the methodology cannot be applied directly as described here. The real-world samples are mostly mixtures of different airborne particles, not only microorganisms, but also ash, soot, and mineral dust. Therefore, the preferred methodology is not the described bulk measurement, but rather separate measurement of each particle individually by FTIR microscopy. Unfortunately, implementation of FTIR microspectroscopy is hindered by strong scattering phenomena. Due to scattering there is a large variation between spectra of single grains belonging to the same species [22]. Therefore, likely application of FTIR microspectroscopy in aerobiological studies is highly dependent on the future development of FTIR sampling technology and pre-processing techniques.

### The potential role of infrared spectroscopy in mycology and botany

The invention and development of molecular phylogenetic analysis, based mainly on DNA sequences, has had great impact on fungal systematics. However, as stated before, the drawbacks of this method are its complexity, high costs, and low ratio of known species represented in public nucleotide sequence databases. Moreover, one worrying result of molecular phylogeny is that lineages often do not correlate well with phenotypic features [38,39]. Taxonomic delimitation of fungi is hampered by the fact that genetic isolation precedes reproductive isolation, while morphological differentiation comes last [40]. Hence, delimitation based on phylogenetic studies often leads to morphologically undistinguishable “cryptic species”. Classical morphological taxonomy cannot distinguish cryptic species and has the other disadvantages, such as need for highly educated and experienced scientists with access to extensive taxonomic literature. Therefore, biochemical analysis of fungi by FTIR spectroscopy of spores could have broader implementation in mycological studies, beyond the scope of aeroallergen monitoring. It could provide economical, reliable and timely methodologies for improving fungal taxonomy, as well as for fungal identification and monitoring. As stated previously, implementation of FTIR in aeroallergen monitoring requires development of an air-sampling system for processing of bioparticles that is compatible with FTIR system. On the other hand, in biological studies FTIR can be immediately implemented as described here, by measurement of pollen and spores collected directly from flowers and sporocarps.

In our previous study on pollen grains it has been found that FTIR spectroscopy enables measurement of phenotypic plasticity of plants by the detection of inter-annual variations within the populations [27]. It is reasonable to assume that complementary processes are present and probably measurable in fungal spores as well. Fungi make one of the major clades of life with over 5 million species that have been estimated to exist in nature [41]. They play essential ecological roles in ecosystems, such as carbon cycling and mutualism in the form of mycorrhizae, lichenization and endophytes. Therefore, monitoring of fungal-environment interactions is highly relevant, particularly the effect of climate change on fungal communities. Since spores allow fungi to endure unfavourable conditions and to colonize new environments, chemical characterization of spores by FTIR spectroscopy could be a valuable tool in monitoring and management of terrestrial ecosystems.

## Supporting Information

S1 File Contains the following files: Table A. List of analyzed fungal spores. **Table B.** List of analyzed plant pollens. **Fig. A.** PCA plot of IR spectral data set of fresh spores and pollen.
(PDF)
