# Supplementary material for: Characterizing Aeroallergens by Infrared Spectroscopy of Fungal Spores and Pollen
Source: PLoS One. 2015 Apr 13;10(4):e0124240. doi: 10.1371/journal.pone.0124240 (PMC4395086; doi:10.1371/journal.pone.0124240)

Supporting Information for:

Characterizing aeroallergens by infrared spectroscopy of fungal spores and pollen

Boris Zimmermann[1], Zdenko Tkalčec[2], Armin Mešić[2], Achim Kohler[1]

[1] Department of Mathematical Sciences and Technology, Norwegian University of Life Sciences, Ås, Norway.

[2] Division for Marine and Environmental Research, Ruđer Bošković Institute, Zagreb, Croatia.

Table of Contents | Page

Table A. List of analyzed fungal spores 2
Table B. List of analyzed plant pollens 4
Figure A. PCA plot of IR spectral data set of fresh spores and pollen 7

Table A. List of analyzed fungal spores (part I)

SPECIES	SAMPLE	LOCATION	DATE	SAMPLED BY:	DETERMINED BY:
Lycoperdon perlatum	CNF 1/2250	Croatia, Mljet isl., S of Sobra	8 Dec 1999	N. Hajdić	Z. Tkalčec & A. Mešić
	CNF 1/3505	Croatia, Medvednica mt., S of Kraljičin zdenac	12 Sep 2004	J. Franc	Z. Tkalčec & A. Mešić
	CNF 1/5960	Croatia, Southern Dalmatia, near Zaton	29 Nov 1984	I. Focht	Z. Tkalčec & A. Mešić
	CNF 1/5979	Croatia, Medvednica mt., Puntijarka area	26 Aug 2010	Z. Tkalčec	Z. Tkalčec & A. Mešić
	CNF 1/5980	Croatia, Medvednica mt., Puntijarka area	26 Aug 2010	Z. Tkalčec	Z. Tkalčec & A. Mešić
	CNF 1/5981	Croatia, Medvednica mt., Puntijarka area	26 Aug 2010	Z. Tkalčec	Z. Tkalčec & A. Mešić
	CNF 6/364	Slovenia, Kranj area, near Zgornja Bela	26 Oct 1968	M. Tortić	V. Demoulin
	CNF 6/365	Slovenia, Kamnik area, near Volčji Potok	19 Sep 1971	M. Tortić	V. Demoulin
	CNF 6/389	Belgium, Volmersberg near St. Vith	30 Sep 1965	V. Demoulin	V. Demoulin
	CNF 6/426	Croatia, Zagreb	7 Oct 1962	M. Tortić	M. Tortić
	CNF 6/428	Croatia, Korenica area, Laudonov Gaj forest	7 Oct 1965	M. Tortić	M. Tortić, J.T. Palmer & V. Demoulin
Lycoperdon pyriforme	CNF 1/2989	Croatia, Dalmatia, Makarska area, near Tučepi	20 Jun 2002	A. Sudar & I. Kušan	Z. Tkalčec & A. Mešić
	CNF 1/4113	Croatia, Sjeverni Velebit Nat. Park, Štirovača area	21 Sep 2006	Z. Tkalčec	Z. Tkalčec & A. Mešić
	CNF 1/4771	Croatia, Gorski kotar, Stara Sušica near Ravna Gora	17 Oct 2007	B. Pavlek	Z. Tkalčec & A. Mešić
	CNF 1/6047	Croatia, Medvednica mt., near Rauhova lugarnica mt. hut	10 Oct 2010	Z. Tkalčec	Z. Tkalčec & A. Mešić
	CNF 1/6048	Croatia, Medvednica mt., near Rauhova lugarnica mt. hut	10 Oct 2010	Z. Tkalčec	Z. Tkalčec & A. Mešić
	CNF 6/367	Croatia, Zagreb, Maksimir park	26 Nov 1961	M. Tortić	M. Tortić & V. Demoulin
	CNF 6/368	Croatia, Gorski kotar, Crni Lug area, near Bela Vodica	30 Sep 1962	M. Tortić	M. Tortić & V. Demoulin
	CNF 6/375	Croatia, Lika region, Otočac area, S of Vrhovine	24 Oct 1965	M. Tortić	M. Tortić & J.T. Palmer
	CNF 6/376	Serbia, Goč mt. near Kraljevo, Dobre Vode tourist resort	1966	M. Jelić	J.T. Palmer
Scleroderma areolatum	CNF 1/1888	Croatia, Zagreb, Črnomerec	16 Oct 2000	Đ. Tkalčec	Z. Tkalčec & A. Mešić
	CNF 1/1960	Croatia, Zagreb, Črnomerec	22 Oct 2000	Z. Tkalčec & A. Mešić	Z. Tkalčec & A. Mešić
	CNF 1/3457	Croatia, Zagreb, Jelenovac park forest	25 Jul 2004	Z. Tkalčec	Z. Tkalčec & A. Mešić
	CNF 1/3476	Croatia, Zagreb, Prekrižje area	22 Aug 2004	J. Franc	Z. Tkalčec & A. Mešić
	CNF 1/5994	Croatia, Zagreb, Ksaver area	2 Sep 2010	Z. Tkalčec & A. Mešić	Z. Tkalčec & A. Mešić
	CNF 1/6007	Croatia, Zagreb, Maksimir park	5 Sep 2010	Z. Tkalčec	Z. Tkalčec & A. Mešić
	CNF 1/6008	Croatia, Zagreb, Maksimir park	5 Sep 2010	Z. Tkalčec	Z. Tkalčec & A. Mešić
	CNF 1/6012	Croatia, Zagreb, Jelenovac park forest	4 Sep 2010	A. Mešić	Z. Tkalčec & A. Mešić
	CNF 6/400	Poland, Białowieża National Park	6 Sep 1966	V. Demoulin	V. Demoulin
Scleroderma citrinum	CNF 1/5996	Croatia, Zaprešić area, NE of Dubravica	4 Sep 2010	Z. Tkalčec	Z. Tkalčec & A. Mešić
	CNF 1/5997	Croatia, Zaprešić area, NE of Dubravica	4 Sep 2010	Z. Tkalčec	Z. Tkalčec & A. Mešić
	CNF 6/399	Great Britain, Derbyshire Peak District, Win Hill	18 Aug 1968	M. Tortić & J.T. Palmer	M. Tortić & J.T. Palmer
	CNF 6/406	Slovenia, between Poljčane & Rogaška Slatina	Aug 1970	V. Demoulin	V. Demoulin
	CNF 6/407	Slovenia, Ljubljana, Golovec forest	26 Oct 1968	M. Tortić	M. Tortić & V. Demoulin

Table A. List of analyzed fungal spores (part II)

SPECIES	SAMPLE	LOCATION	DATE	SAMPLED BY:	DETERMINED BY:
Geastrum triplex	CNF 1/12	Croatia, Medvednica mt., near Glavica mt. hut	23 Mar 1985	Č. Herman	Z. Tkalčec & A. Mešić
	CNF 1/1424	Croatia, Čakovec area, S of Totovec	29 Apr 2000	V. Burić	Z. Tkalčec & A. Mešić
	CNF 1/3035	Croatia, Gorski kotar, Mrkopalj area, NW of Sunger	12 Apr 2003	M. Čerkez	Z. Tkalčec & A. Mešić
	CNF 1/3646	Croatia, Lastovo isl., NE of Uble	18 Mar 2005	D. Mrvoš	Z. Tkalčec & A. Mešić
	CNF 1/3819	Croatia, Plitvička jezera Nat. Park, Čorkova uvala forest reserve	11 Oct 2005	Z. Tkalčec & A. Mešić	Z. Tkalčec & A. Mešić
	CNF 1/6003	Croatia, Žumberak mt., between Plešivica and Prekrižje Plešivičko	4 Sep 2010	Z. Tkalčec	Z. Tkalčec & A. Mešić
	CNF 1/6004	Croatia, Žumberak mt., between Plešivica and Prekrižje Plešivičko	4 Sep 2010	Z. Tkalčec	Z. Tkalčec & A. Mešić
	CNF 1/6017	Croatia, Medvednica mt., Puntijarka area	12 Sep 2010	Z. Tkalčec	Z. Tkalčec & A. Mešić
	CNF 6/447	Slovenia, Kočevski Rog mt., Rajhenavski Rog forest reserve	9 Nov 1977	M. Tortić	M. Tortić
	CNF 6/452	Slovenia, Rogaška Slatina area, near Podčetrtek	8 Dec 1974	M. Tortić	M. Tortić, Z. Tkalčec & A. Mešić
	CNF 6/454	Macedonia, Debar area, near Debarska banja spa	22 Sep 1972	A. Gudeski	M. Tortić
	CNF 6/455	Serbia, Goč mt. near Kraljevo, Dobre Vode tourist resort	10 Oct 1967	M. Tortić & M. Jelić	M. Tortić & M. Jelić
	CNF 6/457	Macedonia, Kožuf mt., near Dudica peak	10 Oct 1967	A. Gudeski	J. T. Palmer
	CNF 6/459	Czech Republic, Prague area, Hostivice, sport park Břve	24 Sep 1966	F. Kotlaba	F. Kotlaba
	CNF 6/460	Belgium, Brugge area, between De Haan and Wenduine	31 Oct 1965	V. Demoulin	V. Demoulin
	CNF 6/461	Croatia, Lika region, Mala Kapela mt., near Babin Potok	24 Oct 1965	M. Tortić	M. Tortić & J. T. Palmer
Geastrum fimbriatum	CNF 1/395	Croatia, Cres isl., near lake Vransko jezero, NW of Vrana	7 Apr 1992	Z. Tkalčec	Z. Tkalčec & A. Mešić
	CNF 1/2164	Croatia, Žumberak mt., between Plešivica and Prekrižje Plešivičko	11 Feb 2001	Z. Tkalčec	Z. Tkalčec & A. Mešić
	CNF 1/2298	Croatia, Ivanščica mt.	26 Aug 2001	T. Slaviček	Z. Tkalčec & A. Mešić
	CNF 1/3309	Croatia, Dalmatia, Zadar area, SE of Kožino	6 Jan 2003	M. Čerkez	Z. Tkalčec & A. Mešić
	CNF 1/5972	Croatia, Gorski kotar, near Moravice	10 Sep 1978	I. Focht	Z. Tkalčec & A. Mešić
	CNF 1/5998	Croatia, Žumberak mt., between Plešivica and Prekrižje Plešivičko	4 Sep 2010	Z. Tkalčec	Z. Tkalčec & A. Mešić
	CNF 1/6001	Croatia, Žumberak mt., between Plešivica and Prekrižje Plešivičko	4 Sep 2010	Z. Tkalčec	Z. Tkalčec & A. Mešić
	CNF 1/6002	Croatia, Žumberak mt., between Plešivica and Prekrižje Plešivičko	4 Sep 2010	Z. Tkalčec	Z. Tkalčec & A. Mešić
	CNF 1/6005	Croatia, Žumberak mt., between Plešivica and Prekrižje Plešivičko	4 Sep 2010	Z. Tkalčec	Z. Tkalčec & A. Mešić
	CNF 5/165	Croatia, Lika region, Ličko Lešće area, near Podkoren	25 Sep 1998	Z. Tkalčec & A. Mešić	Z. Tkalčec & A. Mešić
	CNF 6/466	Croatia, Žumberak mt., near Smerovišće	25 Oct 1970	M. Tortić	M. Tortić
	CNF 6/467	Slovenia, Kranjska Gora area, near Rateče	8 Sep 1969	M. Tortić & J. T. Palmer	M. Tortić & J. T. Palmer
	CNF 6/470	Croatia, Gorski kotar, Crni Lug area, near Bela Vodica	3 Oct 1965	Z. Abramović	M. Tortić & J. T. Palmer
	CNF 6/471	Croatia, Gorski kotar, Risnjak National Park, between Leska and Veliki Risnjak peak	2 Oct 1962	M. Tortić	V. Lindtner
Geastrum melanocephalum	CNF 6/465	Serbia, Vojvodina province, Deliblatska peščara sand reserve, near Devojački Bunar	23 Oct 1967	M. Jelić	M. Jelić
Boletus depilatus	CNF 1/1534	Croatia, Medvednica mt., N of Gračani	16 Jul 2000	V. Burić	Z. Tkalčec & A. Mešić
Agrocybe pediades	CNF 1/1552	Croatia, Zaprešić area, NE of Dubravica	20 Jul 2000	Z. Tkalčec & A. Mešić	Z. Tkalčec & A. Mešić
Amanita citrina	CNF 1/3846	Croatia, Medvednica mt., N of Mikulići	16 Oct 2005	Z. Tkalčec	Z. Tkalčec & A. Mešić
Amanita pachyvolvata	CNF 1/4076	Croatia, Generalski Stol area, N of Grabrk	10 Sep 2006	B. Pavlek	Z. Tkalčec & A. Mešić
Leccinum pseudoscabrum	CNF 1/1282	Croatia, Donja Stubica area, near Golubovec castle	24 Jul 1999	Z. Tkalčec	Z. Tkalčec & A. Mešić

Table B. List of analyzed plant pollens (part I)

ORDER	FAMILY	GENUS	SPECIES	COMMON NAME
Fagales	*Betulaceae*	*Alnus*	*A. incana*	Grey Alder
			A. glutinosa	Black Alder
		Corylus	*C. maxima*	Filbert
			C. avellana	Common Hazel
			C. chinensis	Chinese Hazel
			C. colurna	Turkish Hazel
			C. sieboldiana	Asian Beaked Hazel
		Ostrya	*O. carpinifolia*	European Hop-hornbeam
		Betula	*B. ermanii*	Erman’s Birch
			B. pendula	Silver Birch
			B. papyrifera	Paper Birch
		Carpinus	*C. orientalis*	Oriental Hornbeam
			C. betulus	European Hornbeam
	Fagaceae	*Quercus*	*Q. robur*	Pedunculate Oak
			Q. coccinea	Scarlet Oak
			Q. rubra	Northern Red Oak
			Q. petreae	Sessile Oak
			Q. ilex	Holly Oak
			Q. coccifera	Kermes Oak
			Q. faginea	Portuguese Oak
			Q. cerris	Turkey Oak
			Q. pubescens	Downy Oak
			Q. libani	Lebanon Oak
			Q. shumardii	Shumard’s Oak
			Q. frainetto	Hungarian Oak
		Fagus	*F. sylvatica*	European Beech

Table B. List of analyzed plant pollens (part II)

ORDER	FAMILY	GENUS	SPECIES	COMMON NAME
Poales	*Cyperaceae*	*Holoschoenus*	*H. romanus*	Round-headed Club-rush
		Carex	*C. dipsacea*	New Zealand Sedge
			C. pendula	Pendulous Sedge
			C. muskingumensis	Muskingum Sedge
			C. flacca	Blue Sedge
			C. divisa	Divided Sedge
			C. ferruginea	Rusty Sedge
			C. morrowii	Variegata Sedge
			C. riparia	Great Pond-Sedge
			C. ornithopoda	Birdsfoot Sedge
			C. grayi	Gray's Sedge
			C. sylvatica	Forest Sedge
			C. ovalis	Eggbract Sedge
		Cladium	*C. mariscus*	Sawtooth Sedge
		Eleocharis	*E. palustris*	Common Spikerush
	Poaceae	*Secale*	*S. cereale*	Rye
		Alopecurus	*A. pratensis*	Meadow Foxtail
		Brachypodium	*B. retusum*	Mediterranean False-brome
			B. pinnatum	Heath False-brome
		Festuca	*F. filiformis*	Fine-Leaved Sheep's Fescue
			F. ovina	Sheep Fescue
			F. pratensis	Meadow Fescue
			F. drymeja	Mountain Fescue
			F. amethystina	Tufted Fescue
			F. arvernensis	Field Fescue
			F. heterophylla	Various-leaved Fescue
		Melica	*M. altissima*	Siberian Melic
			M. nutans	Mountain Melic
		Poa	*P. badensis*	Baden's Bluegrass
			P. pratensis	Smooth Meadow-grass
			P. nemoralis	Wood Meadow-Grass
			P. compressa	Flattened Meadow-grass
		Anthoxanthum	*A. odoratum*	Sweet Vernal Grass
		Arrhenatherum	*A. elatius*	Tall Oat-grass
		Sesleria	*S. tenuifolia*	Thin-leaved Moor Grass
			S. nitida	Nest Moor Grass
		Dactylis	*D. glomerata*	Orchard Grass
		Bromus	*B. erectus*	Erect Brome
		Briza	*B. media*	Common Quaking Grass
		Lolium	*L. perenne*	Perennial Ryegrass
		Holcus	*H. lanatus*	Common Velvet Grass
		Phalaris	*P. canariensis*	Phalaris canariensis

Table B. List of analyzed plant pollens (part III)

ORDER	FAMILY	GENUS	SPECIES	COMMON NAME
Pinales	*Cupressaceae*	*Platycladus*	*P. orientalis*	Chinese Arborvitae
		Thujopsis	*T. dolabrata*	Thujopsis
		Chamaecyparis	*C. lawsoniana*	Lawson's Cypress
			C. obtusa	Hinoki Cypress
			C. pisifera	Sawara Cypress
		Juniperus	*J. chinensis*	Chinese Juniper
			J. virginiana	Eastern Juniper
			J. sabina	Savin Juniper
			J. phoenicea	Phoenicean Juniper
			J. communis	Common Juniper
			J. oxycedrus	Prickly Juniper
			J. excelsa	Greek Juniper
		Calocedrus	*C. decurrens*	California Incense-cedar
		Cupressus	*C. sempervirens*	Mediterranean Cypress
			C. x leylandii	Leyland Cypress
			C. lusitanica	Mexican Cypress
			C. sargentii	Sargent Cypress
		Cunninghamia	*C. lancelota*	China Fir
		Cryptomeria	*C. japonica*	Japanese Cedar
		Thuja	*T. occidentalis*	Eastern Arborvitae
			T. standishii	Japanese Thuja
		Taxodium	*T. distichum*	Bald Cypress
		Sequoia	*S. sempervirens*	Coast Redwood
		Metasequoia	*M. glyptostroboides*	Dawn Redwood
	Pinaceae	*Tsuga*	*T. canadensis*	Eastern Hemlock
		Cedrus	*C. atlantica*	Atlas Cedar
			C. deodara	Deodar Cedar
		Abies	*A. koreana*	Korean Fir
			A. pinsapo	Spanish Fir
			A. alba	Silver Fir
			A. cephalonica	Greek Fir
		Picea	*P. abies*	Norway Spruce
			P. asperata	Dragon Spruce
			P. omorika	Serbian Spruce
			P. chihuahuana	Chihuahua Spruce
			P. orientalis	Caucasian Spruce
			P. smithiana	Morinda Spruce
			P. pungens	Blue Spruce
		Pinus	*P. pinea*	Stone Pine
			P. mugo	Mountain Pine
			P. sylvestris	Scots Pine
			P. tabuliformis	Chinese Red Pine
			P. banksiana	Jack Pine
			P. pinaster	Maritime Pine
			P. densiflora	Japanese Red Pine
			P. nigra	European Black Pine
			P. ponderosa	Ponderosa Pine
			P. resinosa	Red Pine
			P. wallichiana	Himalayan pine
			P. bungeana	Lacebark Pine
			P. peuce	Macedonian Pine
			P. strobus	Eastern White Pine
			P. heldreichii	Bosnian Pine

Figure A. **a)** PCA plot of IR spectral data set of fresh spores and pollen (three spectra per sample; MSC corrected spectra), with depiction of class: **A** - Agaricomycetes, **M** - Magnoliopsida, **L** - Liliopsida, **P** - Pinopsida. The percent variances for the first five PCs are 57.65, 29.57, 4.68, 2.32 and 1.24.

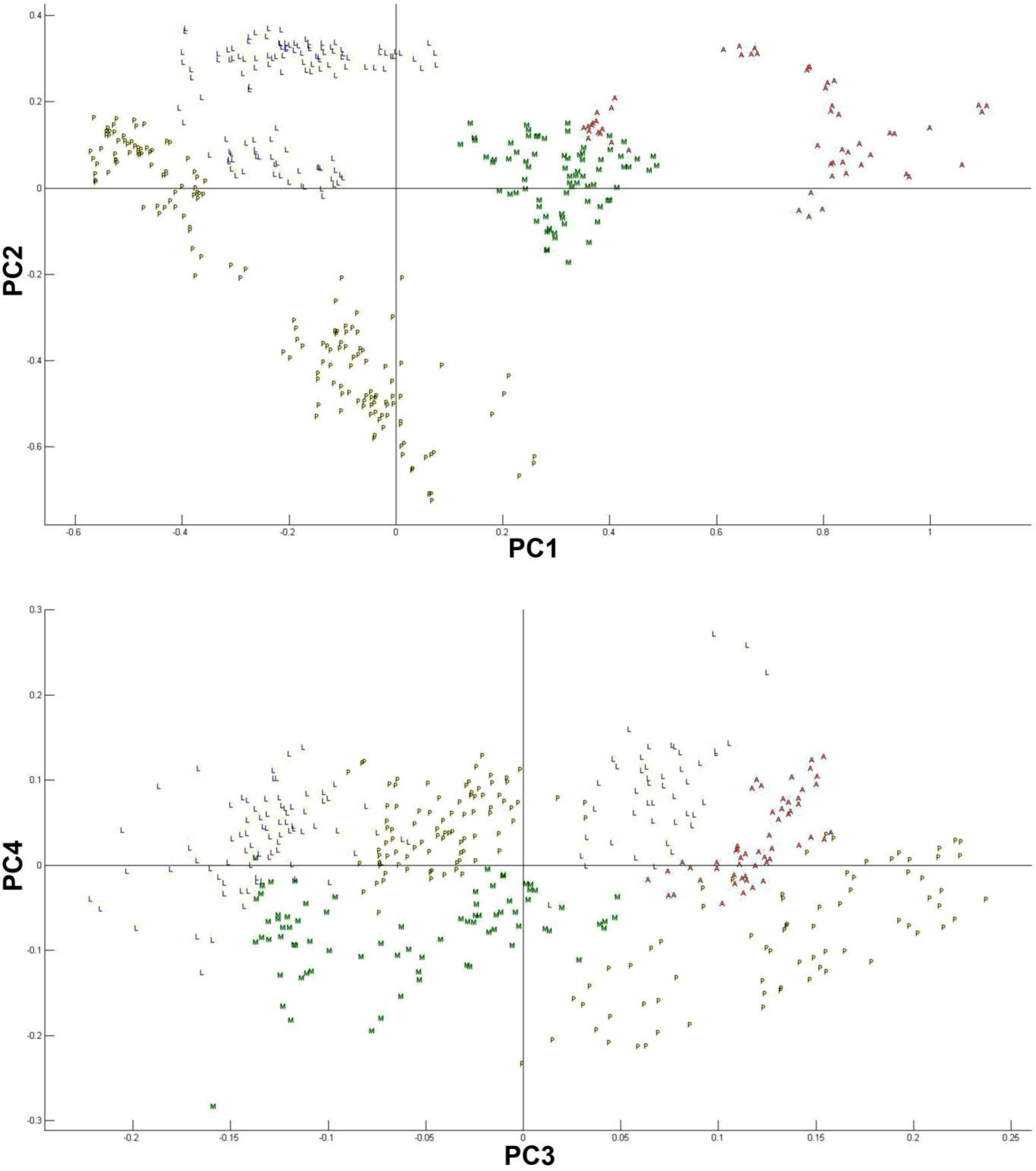

Figure A. **b)** PCA plot of IR spectral data set of fresh spores and pollen (three spectra per sample; MSC corrected spectra), with depiction of familiy: **Cup** - Cupressaceae, **Pin** – Pinaceae; **Cyp** – Cyperaceae, **Poa** – Poaceae, **Bet** – Betulaceae, **Fag** – Fagaceae, **Gea** – Geastraceae, **Scl** – Sclerodermataceae, **Lyc** -Agaricaceae. The percent variances for the first five PCs are 57.65, 29.57, 4.68, 2.32 and 1.24.

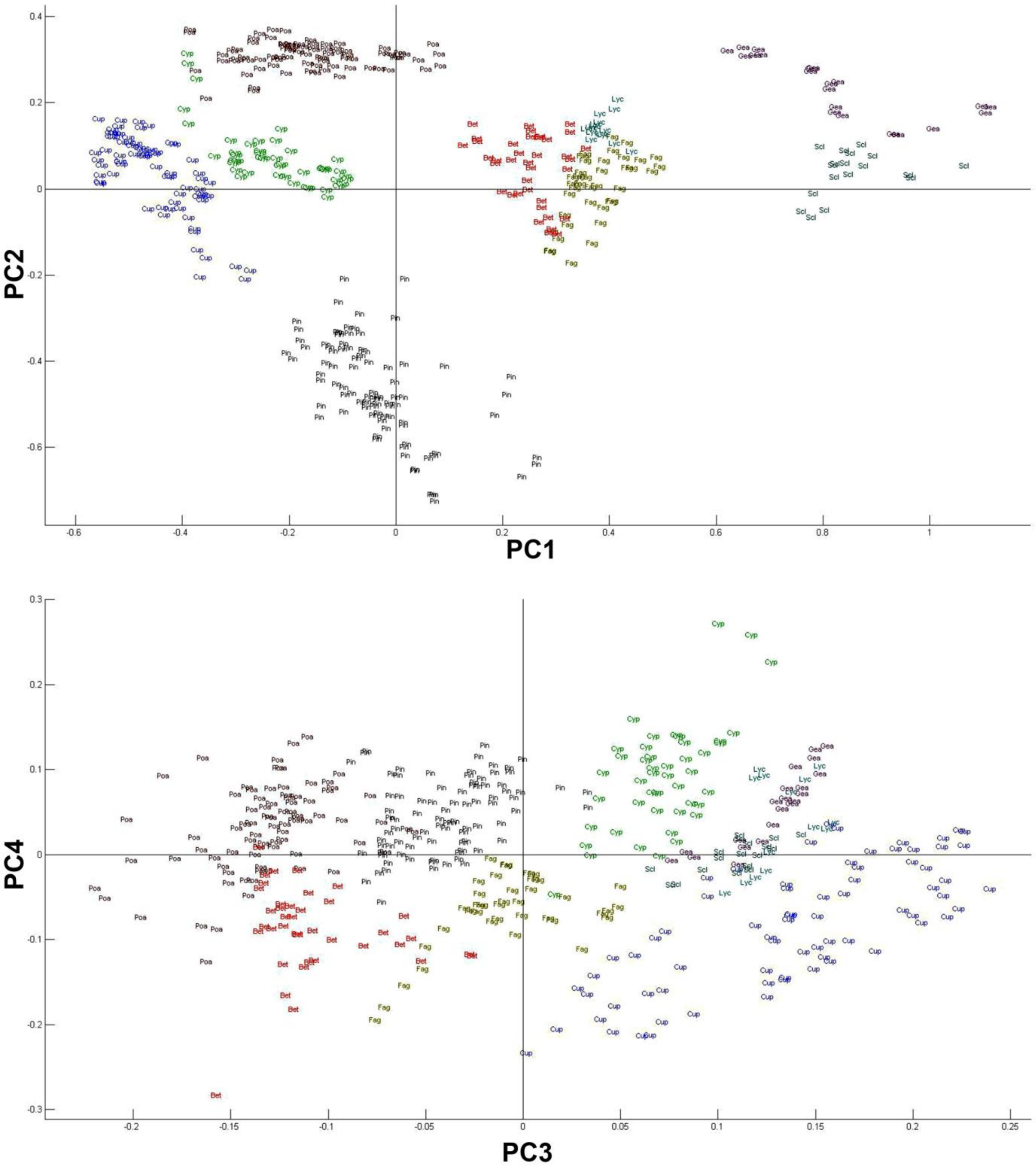

Supplement: S1 File — List of analyzed fungal spores. Table B. List of analyzed plant pollens. Fig. A. PCA plot of IR spectral data set of fresh spores and pollen. (PDF) [file pone.0124240.s001.pdf]
